# Preeclampsia Status Controls Interleukin-6 and Soluble IL-6 Receptor Release from Neutrophils and Endothelial Cells: Relevance to Increased Inflammatory Responses

**DOI:** 10.3390/pathophysiology28020013

**Published:** 2021-04-08

**Authors:** Yuping Wang, Yang Gu, J. Steven Alexander, David F. Lewis

**Affiliations:** 1 Department of Obstetrics and Gynecology, Louisiana State University Health Sciences Center-Shreveport, Shreveport, LA 71130, USA; ygu@lsuhsc.edu (Y.G.); dlewi1@lsuhsc.edu (D.F.L.); 2 Department of Molecular and Cellular Physiology, Louisiana State University Health Sciences Center-Shreveport, Shreveport, LA 71130, USA; jalexa@lsuhsc.edu

**Keywords:** IL-6, sIL-6R, sgp130, neutrophils, endothelial cells, placenta, preeclampsia

## Abstract

Increased neutrophil–endothelial binding and inflammatory responses are significant pathophysiological events in the maternal vascular system in preeclampsia, a hypertensive disorder in human pregnancy. Interleukin 6 (IL-6) and its soluble receptors (soluble IL-6R (sIL-6R) and soluble gp130 (sgp130)) are critical inflammatory mediators. During pregnancy, maternal IL-6 and sgp130 levels were increased, but sIL-6R levels were decreased, in women with preeclampsia compared to normotensive pregnant women. However, little is known about differences in IL-6, sIL-6R, and sgp130 production by neutrophils and endothelial cells between normal pregnancy and preeclampsia. To study this, we isolated neutrophils and cultured human umbilical vein endothelial cells (HUVECs) from normal and preeclamptic pregnancies. Production of IL-6, sIL-6R, and sgp130 was measured. The role of placental factor(s)-mediated neutrophil production of IL-6, sIL-6R, and sgp130 was also determined by pretreating neutrophils with placental conditioned medium generated from placental villous cultures. We found that IL-6 and sgp130 were mainly produced by endothelial cells, while sIL-6R was mainly produced by neutrophils. Endothelial cells from preeclampsia produced significantly more IL-6 and sgp130, and neutrophils from preeclampsia produced significantly less sIL-6R than normal pregnancy cells. Interestingly, production of IL-6, sIL-6R, and sgp130 were time-dependently increased when neutrophils and endothelial cells were co-cultured. We also found that neutrophils from normal pregnancies produced more IL-6, but less sIL-6R, after being primed by preeclamptic-placental conditioned medium. These results demonstrated that neutrophils and endothelial cells have different capacities in producing IL-6, sIL-6R, and sgp130 between normal pregnancy and preeclampsia. These results also provide evidence that the placenta plays a role in inducing neutrophil activation in preeclampsia.

## 1. Introduction

In the vascular system, endothelial cells and circulating neutrophils are major components of the systemic response to inflammation. During pregnancy, an excessive inflammatory phenotype is a central pathophysiological event in the vascular system in preeclampsia [1], a hypertensive, and multi-system disorder unique to human pregnancy. Preeclampsia is characterized by maternal hypertension and proteinuria after 20 weeks of gestation. In preeclampsia, maternal levels of inflammatory mediators, such as inflammatory cytokines interleukin 6 (IL-6), IL-8, and tumor necrosis factor-α (TNFα) [2,3,4], and endothelial adhesion molecules intercellular adhesion molecule (ICAM)-1, vascular cell adhesion molecule-1 (VCAM-1), and P-selectin [5,6] are elevated. Neutrophil adhesion molecules CD11b, CD64, and L-selectin are also significantly increased in women with preeclampsia compared to normotensive pregnant controls [7,8]. All of these findings support the concept of increased systemic inflammatory response in preeclampsia.

IL-6 is an important pleiotropic cytokine that regulates cell growth and differentiation and plays important roles in immune and inflammatory responses [9,10]. IL-6 acts as both a pro-inflammatory and an anti-inflammatory cytokine via IL-6 receptor signals. Membrane receptors for IL-6 are protein complexes consisting of a ligand binding subunit IL-6 receptor (IL-6R) and a signal transducer subunit glycoprotein 130 (gp130). IL-6R and gp130 also have soluble forms, soluble IL-6R (sIL-6R) and soluble gp130 (sgp130), both of which are bioactive. We previously reported that maternal levels of IL-6 and sgp130 are significantly higher in women with preeclampsia than in women with normal pregnancy [11]. We also found that increased IL-6 and sgp130 levels are associated with reduced suppressor of cytokine signaling-3 (SOCS-3) expression in both maternal vessel endothelium and circulating neutrophils in preeclampsia [11]. SOCS-3 is an important cellular anti-inflammatory mediator [12,13,14]. Therefore, increased maternal IL-6 and sgp130 levels may reflect suppression of endogenous anti-inflammatory activity in endothelial cells and neutrophils in preeclampsia. However, little is known about IL-6, sIL-6R, and sgp130 production by endothelial cells and neutrophils in pregnancy. The purpose of the study was to determine if neutrophils and endothelial cells are sources of IL-6 and its soluble receptors in pregnancy and to test our hypothesis of aberrant IL-6, sIL-6R, and sgp130 production by neutrophils and endothelial cells in preeclampsia. We also tested if neutrophil–endothelial interaction influenced IL-6, and its soluble receptor production, and whether neutrophil production of IL-6 can be induced by placental factors in preeclampsia.

## 2. Materials and Methods

### 2.1. Study Samples

Neutrophils were isolated from freshly obtained maternal venous blood from normal and preeclamptic pregnant women. Endothelial cells (HUVEC) were isolated from umbilical cords. Collection of maternal blood and placenta was approved by the Institutional Review Board (IRB) for human research at Louisiana State University Health Sciences Center-Shreveport. Signed consent was obtained. Normal pregnancy was defined as pregnancy with maternal blood pressure <140/90 mmHg, absence of proteinuria, without maternal or medical complications. Preeclampsia was defined as a maternal blood pressure of 140/90 mmHg or higher on two separate readings at least 6 h apart with proteinuria >1 + by dipstick or >300 mg in 24 h urine. No patients had signs of infection. Smokers and patients with pre-rupture of membranes or complicated with diabetes and autoimmune disorders were excluded. Patient demographic data from whom maternal neutrophils were used in the study is shown in Table 1, and from which placenta and umbilical cord used in the study is shown in Table 2.

### 2.2. HUVEC Isolation and Culture

HUVECs were isolated by collagenase digestion as previously described [15]. Isolated endothelial cells were grown in 25 cm^2^ flasks and cultured with endothelial growth medium (EGM) from Lonza Walkersville Inc. (Walkersville, MD, USA). For experiments, P1 cells (first passage) were seeded with 2 × 10^5^ cells per well in 6 well/plates. Cells grew to approximately 5 × 10^5^ cells/per well after 24 h of incubation. After changing medium, cells were then incubated either alone or co-cultured with freshly isolated neutrophils. All cultures were performed in duplicates. Medium was collected at 2 and 6 h of incubation and stored at −70 °C until assayed.

### 2.3. Neutrophil Isolation and Culture

Polymorphonuclear neutrophils (PMN) were isolated immediately from freshly drawn venous blood as previously described [16,17,18]. Briefly, 3% dextran sedimentation and Histopaque density gradient centrifugation was used to isolate neutrophils. Cells were then incubated with lysis buffer in 2 min on ice to remove contaminating red blood cells. In general, this procedure yielded approximately 1–2 × 10^7^ neutrophils from 10 mL of whole blood with 99% viable cells by trypan blue exclusion and 98% purity by acetic acid–crystal violet staining. Freshly isolated neutrophils 2 × 10^6^ cells/well were incubated with EGM in 6 well/plates either alone or co-cultured with endothelial cells (5 × 10^5^ cell/well). Medium was collected at 2 and 6 h after incubation and stored at −70°C until assayed.

### 2.4. Preparation of Placental Conditioned Medium

Placental conditioned medium was prepared by culturing placental villous tissue for 48 h as previously described [18,19]. Briefly, placentas were processed immediately after delivery. Placental villous tissue was gently separated by sterile dissection from different cotyledons, excluding chorionic and basal plates, minced with scalpel blades, and washed repeatedly with phosphate buffered saline to remove blood. Villous explants (500 mg/well in 6 well plate) were then incubated with 5 mL serum free Dulbecco’s Modified Eagle Medium (DMEM, Sigma, St. Louis, MO, USA) for 48 h. Medium samples were collected at the end of incubation as conditioned medium and pooled conditioned media were used for treating isolated neutrophils.

### 2.5. Measurement of IL-6, sIL-6R, and sgp130

Endothelial and neutrophil production of IL-6, sIL-6R, and sgp130 were measured by enzyme-linked immunosorbent assay (ELISA). DuoSet ELISA development kits of IL-6 (DY206), sIL-6R (DY227), and sgp130 (DY228) were purchased from R&D systems (Minneapolis, MN, USA). ELISA assay was performed according to the manufacturer’s instruction. An aliquot of 100 µL medium sample was assayed and all samples were measured in duplicate. The range of a standard curve was 0.5–600 pg/mL for IL-6, 1–1000 pg/mL for sIL-6R, and 10 pg–10 ng/mL for sgp130. Within- and between-assay variations were less than 6% and 8% for all the assays, respectively.

### 2.6. Statistical Analysis

Demographic data are expressed as mean ± SD and data for IL-6, sIL-6R, and sgp130 production are expressed as mean ± SE. Data were analyzed by unpaired *t*-test, paired *t*-test, or ANOVA. Student-Newman-Keuls test was used as a post-hoc test. A probability level of less than 0.05 was considered statistically significant.

## 3. Results

### 3.1. Different Patterns in IL-6, sIL-6R, and sgp130 Production by Endothelial Cells and Neutrophils from Normal Pregnant Women

**Figure 1:**
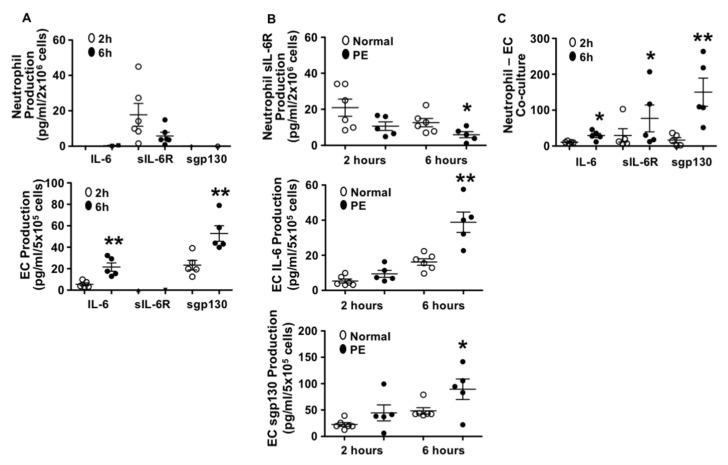
Production of interleukin 6 (IL-6), soluble IL-6R (sIL-6R), and soluble gp130 (sgp130) by neutrophils and endothelial cells from normal and preeclamptic (PE) pregnancies. (**A**) Production of IL-6, sIL-6R, and sgp130 by neutrophils (n = 6) and endothelial cells (n = 5) from normal pregnant women. Open symbol: 2 h of culture, and solid symbol: 6 h of culture, ** *p* < 0.01: 6 h vs. 2 h. (**B**) Comparison of sIL-6R production by neutrophils (normal n = 6; PE n = 5), and IL-6 and sgp130 production by endothelial cells (normal n = 6; PE n = 5) from normal vs. PE pregnancies. Open symbol: normal and solid symbol: PE, * *p* < 0.05 and ** *p* < 0.01: PE vs. normal at 6 h. (**C**) Production of IL-6, sIL-6R, and sgp130 in co-cultured neutrophils and endothelial cells. Freshly isolated neutrophils (2 × 10^6^ cells) were added into cultured endothelial cells (5 × 10^5^ cells/per well in 6 well plate). Both neutrophils and endothelial cells were from normal pregnancy. Results were from five independent experiments. Open symbol: 2 h of culture and solid symbol: 6 h of culture, * *p* < 0.05 and ** *p* < 0.01: 6 h vs. 2 h, respectively.

We first determined IL-6, sIL-6R, and sgp130 production by neutrophils and endothelial cells derived from normal pregnant subjects. In this experiment, neutrophils from six normal pregnant women and HUVECs from five normal placentas were used. Results are shown in Figure 1A. Interestingly, our results showed that neutrophils produced sIL-6R and little IL-6, but not sgp130. In contrast, endothelial cells produced IL-6 and sgp130, but not sIL-6R. These results suggest that under normal conditions, neutrophils probably represent the major source of sIL-6R and endothelial cells are likely the major sources of IL-6 and sgp130. We also noticed that endothelial production of IL-6 and sgp130 was time-dependent, Figure 1A.

### 3.2. Neutrophils Produced Less sIL-6R and Endothelial Cells Produced More IL-6 and sgp130 from Preeclamptic than Those from Normal Pregnancies

To determine if there were differences in IL-6, sIL-6R, and sgp130 production by neutrophils and endothelial cells between normal pregnancy and preeclampsia, neutrophils, and endothelial cells from preeclampsia were also isolated. The culture conditions were the same as that of the cells from normal pregnancies and production of IL-6, sIL-6R, and sgp130 were then determined. We found that compared to the cells from normal pregnancies, neutrophils from preeclampsia produced significantly less sIL-6R, *p* < 0.05 and that endothelial cells from preeclampsia produced significantly more IL-6, *p* < 0.01 and sgp130, *p* < 0.05 (Figure 1B). This increased IL-6 and sgp130 production and decreased sIL-6R production in cells from preeclampsia were also shown to be time-dependent.

### 3.3. Production of IL-6, sIL-6R, and sgp130 in Neutrophil–Endothelial Co-Culture

Increased neutrophil–endothelial adhesion is a phenomenon of increased vascular inflammatory responses. To determine if neutrophil and endothelial production of IL-6, sIL-6R, and sgp130 was affected by cell contact, we designed an experiment in which neutrophils and endothelial cells were co-cultured, and production of IL-6, sIL-6R, and sgp130 were determined. A total of five independent experiments were performed with both neutrophils and endothelial cells derived from normal pregnant subjects. Results are shown in Figure 1C. We found that IL-6, sIL-6R, and sgp130 production were significantly increased in co-cultured cells as compared to either neutrophils or endothelial cells cultured alone (Figure 1A). The increases in IL-6, sIL-6R, and sgp130 production were also time-dependent (Figure 1C).

These results suggest that interaction of neutrophils and endothelial cells promotes neutrophil production of sIL-6R and endothelial production of sgp130. We assume that increased IL-6 production could be derived from both neutrophils and endothelial cells when cells were co-cultured.

### 3.4. Effects of Placenta on Neutrophil Production of IL-6, sIL-6R, and sgp130

**Figure 2:**
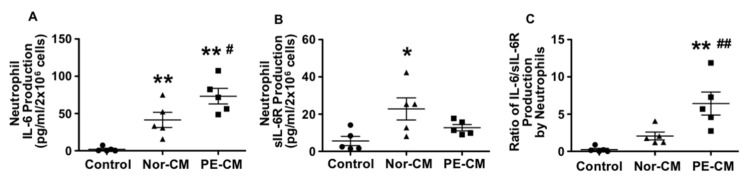
Neutrophil production of IL-6 and sIL-6R after primed by normal and PE placental conditioned medium (CM). Neutrophils produced more IL-6 (**A**) but less sIL-6R (**B**) after being primed by PE-CM compared to cells primed by normal-CM. The ratio of IL-6 to sIL-6R production was significantly increased in cells treated with PE-CM (**C**). * *p* < 0.05 and ** *p* < 0.01: cells primed with CM vs. control; # *p* < 0.05 and ## *p* < 0.01: cells primed with PE-CM vs. normal-CM. Data are represented from five independent experiments.

Neutrophils are activated in women with preeclampsia [18,20,21]. It is believed that during preeclampsia, neutrophils may become activated when they traverse the placental intervillous space in response to trophoblast products, such as reactive oxygen species or inflammatory mediators. To test the role of placental factors in neutrophil IL-6, sIL-6R and sgp130 production, freshly isolated neutrophils from normal pregnancies were treated with either normal or preeclamptic placental conditioned medium for 30 min, and then conditioned medium was removed by centrifugation. Neutrophils were then incubated with fresh EGM for 6 h. Medium was collected and levels of IL-6, sIL-6R, and sgp130 were then measured. As shown in Figure 2A, untreated neutrophils released very little IL-6. However, IL-6 production was significantly increased when neutrophils were exposed to normal conditioned medium and further increased in cells that were exposed to preeclamptic placental conditioned medium as compared to untreated cells, *p* < 0.01.

The sIL-6R production was significantly increased in cells exposed to normal placental conditioned medium, *p* < 0.05, but not in cells exposed to preeclamptic placental conditioned medium, as compared to untreated controls, Figure 2B. Neutrophil release of sgp130 was under detectable in control cells or in cells treated with either normal or preeclamptic placental conditioned medium (data not shown). As a result, the ratio of IL-6/sIL-6R release was about 3-fold greater in neutrophils treated with preeclamptic conditioned medium compared to normal conditioned medium, *p* < 0.01 (Figure 2C). Data represent means from five independent experiments.

## 4. Discussion

In the present study, we investigated differences in IL-6 and its soluble receptor sIL-6R and sgp130 production by neutrophils and endothelial cells from normal and preeclamptic pregnancies. We also determined effects of neutrophil–endothelial interaction on IL-6, sIL-6R, and sgp130 production and placental influences on neutrophil release of IL-6, sIL-6R, and sgp130. Several interesting results were observed. First, we found different patterns in IL-6, sIL-6R, and sgp130 production by neutrophils and endothelial cells under normal (without stimulation) conditions, i.e., neutrophils released measurable sIL-6R and very little IL-6, but not sgp130, whereas endothelial cells released IL-6 and sgp130, but not sIL-6R. These data suggest that neutrophils and endothelial cells may work in concert to control production and release of IL-6 and its soluble receptors. If this is the case in the normal physiological condition, our data suggest that neutrophils are likely a significant source of sIL-6R and that endothelial cells are probably major sources of IL-6 and sgp130 in the circulation. Although the sample size is small in our study, the results are fairly consistent.

**Figure 3:**
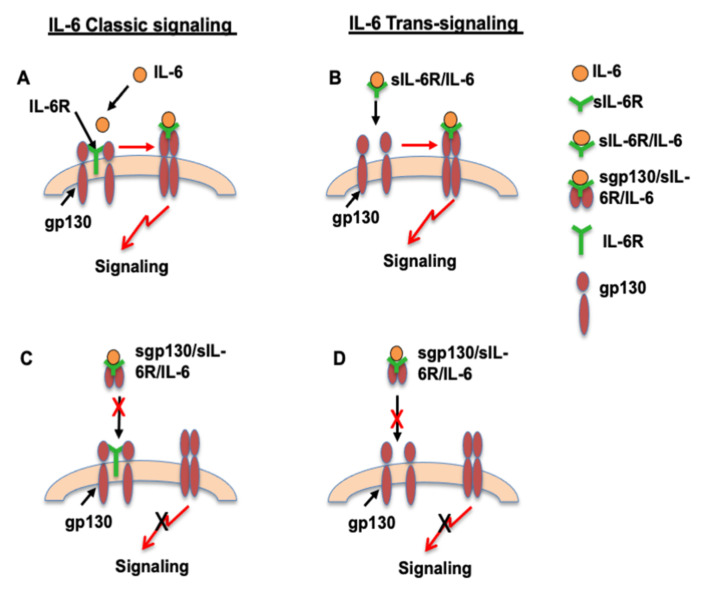
Proposed differences in IL-6 classic signaling and IL-6 trans-signaling in neutrophils and endothelial cells. IL-6 classic signaling in neutrophils (**A**) and trans-signaling in endothelial cells (**B**) in unstimulated condition. A: IL-6 classic signaling in neutrophils. Neutrophils have both IL-6R and gp130. IL-6 binds to IL-6R and then initiates gp130 trans-signaling. B: IL-6 trans-signaling in endothelial cells. Endothelial cells have gp130, but not IL-6R. sIL-6R/IL-6 complex binds to gp130 and then initiates gp130 trans-signaling. C and D: Proposed dysregulation of IL-6 classic signaling in neutrophils (**C**) and trans-signaling in endothelial cells (**D**) in preeclamptic condition. Increased sgp130 production and increased sgp130/sIL-6R/IL-6 complex formation could prevent IL-6 to bind to IL-6R in neutrophils (**C**) and block sIL-6R/IL-6 to bind to gp130 in ECs, as a result, blocks gp130 trans-signaling.

Both IL-6R and gp130 are present in neutrophils [11]. Identification of neutrophils as a source of sIL-6R and endothelial cells as a source of sgp130 is very important in terms of IL-6 signaling in the cardiovascular system. Both sIL-6R and sgp130 are bioactive. IL-6 can bind to IL-6R on cells and to sIL-6R in the circulation. As illustrated in Figure 3A of the IL-6 classic signaling pathway, IL-6 binds to and stimulates cells that express IL-6R [22], such as in neutrophils, while cells that express gp130, but not IL-6R, are unresponsive to IL-6 alone [22], but could respond to sIL-6R/IL-6 complex and subsequently initiate IL-6 trans-signaling as illustrated in Figure 3B. This might be the case in endothelial cells. It is believed that sIL-6R is required for endothelial responsiveness to IL-6 [11,23]. In contrast, sgp130 is considered an endogenous antagonist to sIL-6R/IL-6, because sgp130 binds to sIL-6R/IL-6 and then forms sgp130/sIL-6R/IL-6 complex, which could block both IL-6 classic signaling and sIL-6R/IL-6-mediated IL-6 trans-signaling. Therefore, sgp130 plays a critical role in controlling neutrophil responses to IL-6 or endothelial responses to sIL-6R/IL-6 by determining the ligand/receptor complexes formed and the induced downstream signaling effects, as illustrated in Figure 3C,D.

Second, we found that neutrophils from women with preeclampsia produced significantly less sIL-6R, and endothelial cells from women with preeclampsia produced significantly more IL-6 and sgp130 than cells from normal pregnant subjects. These results are consistent with our previous findings of increased maternal IL-6 and sgp130 levels and an increased ratio of sgp130/sIL-6R/IL-6 in women with preeclampsia compared to normal pregnant controls [11]. Higher sIL-6R levels have protective effects on the cardiovascular system, because higher serum levels of sIL-6R were found to link to a lower risk of coronary heart diseases [24,25]. Therefore, reduced neutrophil production of sIL-6R may likely reflect diminished neutrophil anti-inflammatory activities in preeclampsia. In addition, we also noticed that measurable sIL-6R levels were lower at 6 h than at 2 h in culture in both normal and preeclamptic cells. The reason for this phenomenon is not known, but could reflect sIL-6R/IL-6 or sgp130/sIl-6R/IL-6 complex formation in vitro, which might explain for little IL-6 or unmeasurable sgp130 in neutrophil culture. Nonetheless, reduced neutrophil sIL-6R production was consistent in preeclamptic compared to normal cells.

The phenomenon of increased IL-6 and sgp130 production by endothelial cells is even more interesting in preeclampsia. IL-6 is an important pro-inflammatory cytokine that regulates both acute and chronic inflammatory responses and has distinctive roles in driving inflammatory processes, autoimmunity, and endothelial cell dysfunction. IL-6 has also been identified as an independent risk factor for cardiovascular disease [26,27]. Because sgp130 binds to sIL-6R/IL-6 and blocks sIL-6R/IL-6 trans-signaling-induced endogenous anti-inflammatory activity in endothelial cells (such as SOCS-3 induction) [14,28,29,30], increased sgp130 production could be considered an indicator of reduced anti-inflammatory activity in preeclampsia.

In our study, HUVECs were used as an endothelial model. Although HUVECs are not maternal in origin, they exhibit many phenomena of endothelial activation/dysfunction found in the maternal systemic vasculature in preeclampsia, such as increased endothelial adhesion molecule and protease activating receptor expression [15], reduced endothelial junction molecule and endothelial nitric oxide synthase (eNOS) expression [31], and altered miRNA expression [32]. Therefore, we believe that results obtained using HUVECs could reflect inflammatory phenotypic changes that occur in preeclampsia. This concept is well supported by our findings of increased IL-6 and sgp130 production by preeclamptic HUVECs and elevated maternal levels of IL-6 and sgp130 in women with preeclampsia [11].

Next, we determined if interaction of neutrophils with endothelial cells promotes IL-6, sIL-6R, and sgp130 production in our neutrophil–endothelial co-culture model. Our results showed that production of IL-6, sIL-6R, and sgp130 were all increased when neutrophils and endothelial cells were co-cultured compared to either neutrophils or endothelial cells that were cultured separately. In our study, although neutrophils and endothelial cells were not from the same donor, production of IL-6, sIL-6R, and sgp130 were measured from the same set of neutrophils and endothelial cells in each independent co-culture experiment. Increased sIL-6R production likely reflects neutrophil activation, and increased sgp130 production could be due to endothelial activation in the co-culture system. In regards to IL-6, we believe that IL-6 was produced by not only endothelial cells, but also by activated neutrophils when neutrophils contacted endothelial cells.

Finally, in pregnancy, maternal neutrophils constantly travel between the maternal systemic vascular system and the placental intervillous space. Therefore, we believe that the placenta has a significant impact on neutrophil activation in preeclampsia. To further test the role of placenta-mediated neutrophil activation and to determine the ability of neutrophils to generate IL-6, sIL-6R, and sgp130, freshly isolated neutrophils were treated with placental conditioned medium and then neutrophil production of IL-6, sIL-6R, and sgp130 measured after incubation. Our results confirmed that neutrophils produce IL-6 and sIL-6R, but not sgp130. Our results further showed that neutrophils produced significantly more IL-6 when cells were primed by preeclamptic placental conditioned medium compared to normal placental conditioned medium. In comparison, neutrophils produced significantly more sIL-6R when cells were exposed to normal placental conditioned medium, but not by cells exposed to preeclamptic placental conditioned medium. As result, the ratio of IL-6 to sIL-6R production was significantly increased in neutrophils treated with preeclamptic placental conditioned medium. These results support a model where placental-derived factors promote neutrophil activation in preeclampsia. Our data also support prior findings reported by Mellembakken et al. [33], in which these investigators found that CD11a, CD11b, and CD11c expression was significantly higher in neutrophils obtained from uterine veins than those from antecubital veins in women with preeclampsia. This phenomenon was not seen in neutrophils from normal pregnancies [33]. Taken together, these findings indicate that neutrophil activation can take place when cells circulate through placental intervillous space in preeclampsia.

In summary, endothelial and neutrophil activation/dysfunction associated with increased inflammatory response are significant vascular events in preeclampsia. IL-6 and its receptor trans-signaling represent an important cytokine axis in the pathogenesis of inflammation-associated disorders, including cardiovascular diseases and diabetes [34,35]. Maternal IL-6 and sgp130 are increased in women with preeclampsia [11]. To our knowledge, this is the first study to determine differences in IL-6 and its soluble receptor sIL-6R and sgp130 production in neutrophils and endothelial cells from normal pregnancy and preeclampsia. As proposed in Figure 3C,D, abnormal production of sIL-6R and sgp130 could lead to aberrant IL-6 signaling and contribute to elevated inflammatory responses in preeclampsia. Consequently, the findings of differences in sIL-6R and sgp130 production between neutrophils and endothelial cells and the role of placental factor(s) as an engine of neutrophil activation provide strong additional evidence that preeclampsia status controls IL-6 and its soluble receptor release by neutrophils and endothelial cells. The potential role of circulating sIL-6R and sgp130, and their ratio to IL-6 as biomarkers of increased inflammatory response in preeclampsia or cardiovascular diseases, warrants further investigation.

## Figures and Tables

**Table 1:** Demographic data for maternal neutrophils used in the study.

Variable	Normal (n = 13)	Preeclampsia (n = 5)	*p* Value
Maternal Age (years)	23 ± 5	24 ± 4	0.6822
Racial Status			
White	1	2	ND
Black	12	2	ND
BMI	30 ± 6	40 ± 7	0.0088
Blood Pressure			
Systolic	119 ± 13	179 ± 11	<0.0001
Diastolic	75 ± 9	109 ± 3	<0.0101
Primigravida	10	3	ND
Gestational Age (weeks^+days^)			
at blood draw	32^+5^ ± 4^+5^	29^+5^ ± 2^+1^	0.2401
at delivery	39^+5^ ± 1^+0^	33^+0^ ± 3^+1^	0.0023
Delivery Mode			
Vaginal	12	1	ND
C-section	1	4	ND

Data are expressed as mean ± SD. ND: not determined. BMI = body mass index.

**Table 2:** Demographic data for placentas and umbilical cords used in this study.

Variable	Normal (n = 10)	Preeclampsia (n = 11)	*p* Value
Maternal Age (years)	27 ± 7	23 ± 5	0.1818
Racial Status			
White	2	1	ND
Black	7	10	ND
Other	1	0	ND
BMI	31 ± 8	35 ± 9	0.285
Blood Pressure (mmHg)			
Systolic	114 ± 11	165 ± 12	<0.0001
Diastolic	70 ± 11	101 ± 8	<0.0001
Primigravida	5	6	ND
Gestational Age (weeks^+days^)	39^+0^ ± 1^+1^	35^+0^ ± 3^+5^	0.0052
Delivery Mode			
Vaginal	6	5	ND
C-section	4	6	ND

Data are expressed as mean ± SD. ND: not determined.

## Data Availability

Data are available upon request.
